# Characterization of Two Key Flavonoid 3-*O*-Glycosyltransferases Involved in the Formation of Flower Color in *Rhododendron Delavayi*

**DOI:** 10.3389/fpls.2022.863482

**Published:** 2022-05-16

**Authors:** Wei Sun, Shiyu Sun, Hui Xu, Yuhan Wang, Yiran Chen, Xiaorong Xu, Yin Yi, Zhigang Ju

**Affiliations:** ^1^Key Laboratory of State Forestry Administration on Biodiversity Conservation in Karst Mountain Area of Southwest of China, School of Life Science, Guizhou Normal University, Guiyang, China; ^2^Pharmacy College, Guizhou University of Traditional Chinese Medicine, Guiyang, China

**Keywords:** flower color, anthocyanin, flavonoid 3-*O*-glycosyltransferases, enzyme activity, *Rhododendron delavayi*

## Abstract

Flower color, largely determined by anthocyanin, is one of the most important ornamental values of *Rhododendron delavayi*. However, scant information of anthocyanin biosynthesis has been reported in *R. delavayi*. We found that anthocyanidin 3-*O*-glycosides were the predominant anthocyanins detected in *R. delavayi* flowers accounting for 93.68–96.31% of the total anthocyanins during its development, which indicated the key role of flavonoid 3-*O*-glycosyltransferase (3GT) on *R. delavayi* flower color formation. Subsequently, based on correlation analysis between anthocyanins accumulation and *Rd3GTs* expressions during flower development, *Rd3GT1* and *Rd3GT6* were preliminarily identified as the pivotal *3GT* genes involved in the formation of color of *R. delavayi* flower. Tissue-specific expressions of *Rd3GT1* and *Rd3GT6* were examined, and their function as 3GT *in vivo* was confirmed through introducing into *Arabidopsis UGT78D2* mutant and *Nicotiana tabacum* plants. Furthermore, biochemical characterizations showed that both *Rd3GT1* and *Rd3GT6* could catalyze the addition of UDP-sugar to the 3-OH of anthocyanidin, and preferred UDP-Gal as their sugar donor and cyanidin as the most efficient substrate. This study not only provides insights into the biosynthesis of anthocyanin in *R. delavayi*, but also makes contribution to understand the mechanisms of its flower color formation.

## Introduction

In addition to proteins, fats, and carbohydrates, plants also produce a great deal of additional molecules, which are known as secondary metabolites, including alkaloids, terpenoids, and phenolics (Chuan et al., 2018). Flavonoids, a big cluster of polyphenolic products, occur widely in plants with various biological functions such as pigments, feeding deterrents, antimicrobial agents, chemical messengers, UV protectants, auxins transporters as well as cell cycle inhibitors (Galeotti et al., 2008; Ferreyra et al., 2012). Based on their basic skeleton, flavonoids can be classified into flavonols, flavones, flavanols, flavanones, isoflavones, proanthocyanidins, and anthocyanins (Charles et al., 2010). Among them, anthocyanins are major pigments responsible for the formation of orange, pink, red, purple, and blue colors of many flowers, fruits, and vegetables (Alan et al., 2021; Giuseppe et al., 2021). In addition to this, anthocyanins have also been recognized for their biological benefits on human health. For example, dietary intake of anthocyanin-rich food can reduce the risk of dementia in humans (Commenges et al., 2000). An experimental study on Alzheimer's disease suggests that anthocyanin derivatives may play positive effects on improving cognitive function and neurological resilience (Wang et al., 2008).

The biosynthetic pathway leading to anthocyanin has been well-characterized in many plant species such as *Arabidopsis thaliana, Zea mays*, and *Petunia* × *hybrid* (Winkel-Shirley, 2001; Mandeep et al., 2011; Tohge et al., 2016). Most of the enzymes, including chalcone synthase (CHS), chalcone isomerase (CHI), flavone 3-hydroxylase (F3H), flavonoid 3′-hydroxylase (F3′H), flavonol synthase (FLS), dihydroflavonol 4-reductase (DFR), and anthocyanidin synthase (ANS) related to the biosynthesis of anthocyanin, have been isolated and functionally identified (Sara et al., 2020). But the enzymes involved in the terminal modification (glycosylation, methylation, and acylation) of anthocyanins are less understood, although they are commercially significant for metabolic engineering of flavonoid production (Forkmann and Martens, 2001). Moreover, these modifications can enrich the variety of end products that possess diverse bioactive effects. For instance, over 500 different anthocyanins have been reported in the literature despite the fact that only six chromophore forms (aglycones) existed in anthocyanins. Related studies have also demonstrated that a great part of this diversity is owing to the attachment of different kinds and quantities of sugar moieties at different positions of anthocyanidin, which was called glycosylation (Sonia et al., 2010).

Glycosylation is one of the most extensive modifications that fulfill multifarious functions during plant metabolism, such as an increase in the solubility and stability of acceptor, form glycogen for energy storage, synthesize oligosaccharides at cell surface, detoxify xenobiotics, as well as participate in hormonal homeostasis (Coutinho et al., 2003; Jae et al., 2008). Glycosylation of anthocyanidin is catalyzed by uridine diphosphate glycosyltransferases (UGTs), which transfer a carbohydrate usually from UDP-sugar to a wide range of low-molecular-weight acceptors (Joe et al., 2001). PSPG (Plant Secondary Product Glycosyltransferase) box, a conserved 44-residue motif at C-terminal end, is known as the typical of all UGTs. Several conserved residues in this UGT-defining sequence are found to interact with sugar donor (Gachon et al., 2005; Le et al., 2016). In plant UGTs, UDP-glucose is regarded as the most favored sugar donor, while other similar UDP-sugars, including UDP-galactose, UDP-rhamnose, UDP-xylose, and UDP-arabinose, are also encountered (Yun et al., 2020). The sugar donor preference of UGTs is promiscuous, for example, UGTs from *Vitis Vinifera* and *Fragaria*×*ananassa* can only use UDP-glucose, UCGalT1 from *Daucus carota* L. accepts UDP-galactose only, but 3GT from *Scutellaria baicalensis* transfers at least four kinds of sugars to flavonoids (Christopher et al., 1998; Markus et al., 2008; Zhi-Sheng et al., 2016; Kai et al., 2018; Zilong et al., 2019). At the same time, crystal structures and specific mutagenesis analysis have proved that the last residue in the PSPG box, Arg350, as well as amino acid residues forming hydrogen bonds all play a decisive role on the sugar donor specificity. No conserved amino acid residues have been identified as the general determinants of sugar donor specificity of UGTs (Modolo et al., 2007; Yun et al., 2020).

In addition, glycosyltransferases also have selective substrate specificities as well as regiospecificity. For instance, flavonoid 3-*O*-glycosyltransferases from *Arabidopsis* and petunia can traffic sugar donors only to 3-position of flavonols and anthocyanidins, respectively (Mami et al., 2002; Takayuki et al., 2005). Meanwhile, based on the regiospecificity for substrates, glycosyltransferases can be divided into flavonoid 3-*O*-glycosyltransferases, flavonoid 5-*O*-glycosyltransferases, flavonoid 7-*O*-glycosyltransferases, and so on (Zhao et al., 2012). Of these glycosyltransferases, flavonoid 3-*O*-glycosyltransferases are the ones that are best-studied, as flavonoid 3-*O*-glycosides are the most popular phenolic compounds in plants. The gene encoding flavonoid 3-*O*-glycosyltransferase was first isolated in maize and later characterized at the molecular level in many other plant species (Ralston et al., 1988; Katayama-Ikegami et al., 2020). But for the catalytic mechanism of 3GT, there are still many unsolved problems. Therefore, characterization and mechanistic study of 3GTs from more different plants would help to resolve this complex task.

**Figure 1:**
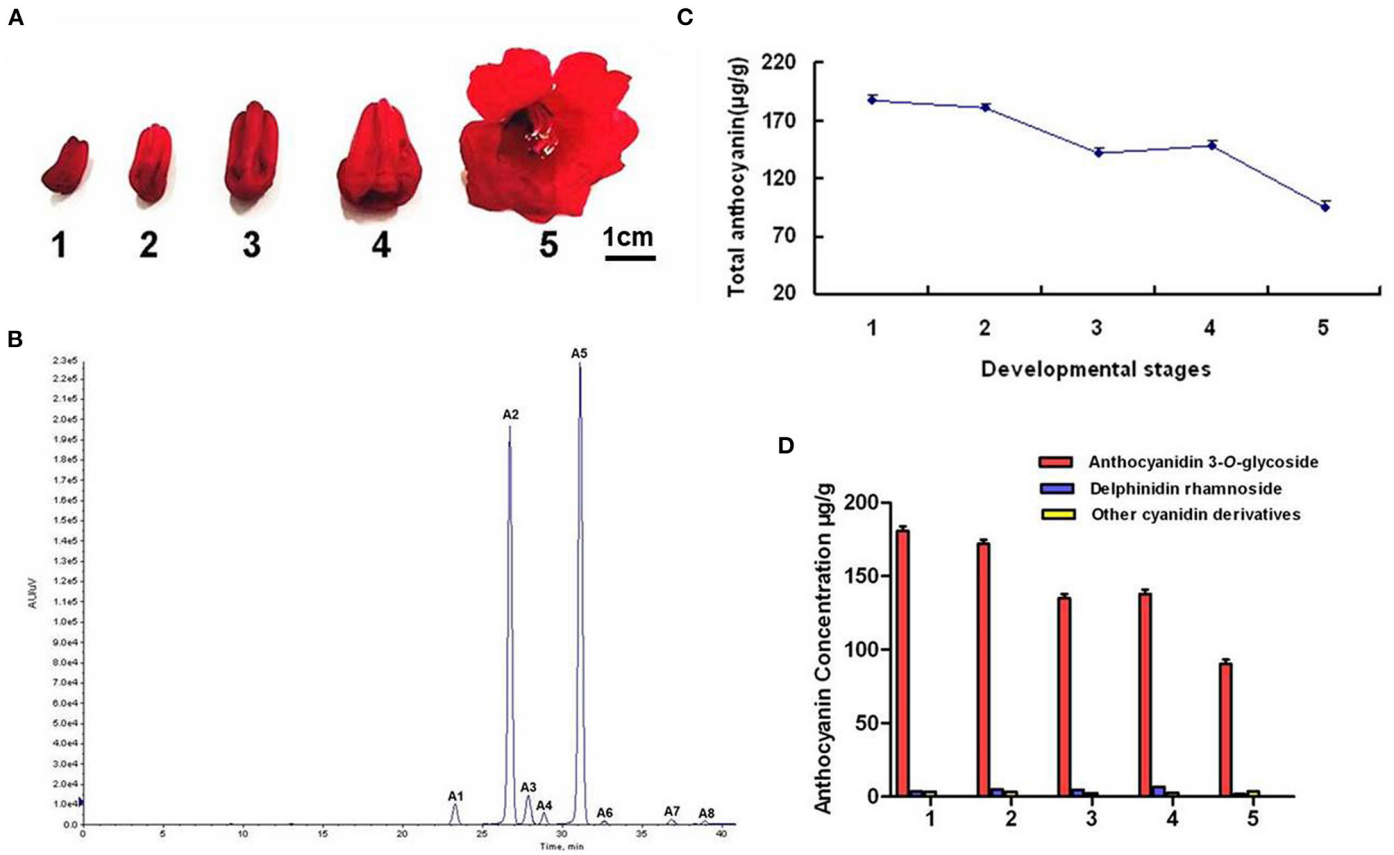
Anthocyanin component analyses in *R. delavayi* flower. **(A)** The phenotypes of different development stage. **(B)** Total anthocyanin profile at different developmental stages. **(C)** HPLC profiles of anthocyanin. A1, delphinidin 3-*O*-galacoside; A2, cyanidin 3-*O*-galacoside; A3, delphinidin rhamnoside; A4, cyanidin 3-*O*-glucoside; A5, cyanidin 3-*O*-arabinoside; A6/A7/A8, other cyanidin derivatives. **(D)** The contents of different anthocyanin at five developmental stages.

*Rhododendron delavayi* (*R. delavayi*) is one of the most famous flowering shrubs. Because of colorful flowers and high horticultural values, it has been widely used in landscape greening (Zhang et al., 2017). Nevertheless, the key enzyme, flavonoid 3-*O*-glycosyltransferase critical for flower color formation, has not been cloned and characterized from *R. delavayi* (Yuan et al., 2021). In the current study, anthocyanins were first identified and quantified at five flower developmental stages, and the anthocyanin biosynthetic pathway in *R. delavayi* flowers was drawn based on these results (Figure 1). Meanwhile, quantitative results showed that anthocyanidin 3-*O*-glycoside was the most abundant during flower development, which accounted for 93.68–96.31% of the total anthocyanins, indicating the importance of *Rd3GTs* for *R. delavayi* flower color formation. Thus, we analyzed the transcriptome data of *R. delavayi* to search for *Rd3GTs*, and six genes with complete open reading frames (ORFs) were obtained. Then, according to the correlation analysis between the anthocyanin accumulation and expression profiles of *Rd3GTs* at different flower developmental stages, two *Rd3GT* genes (*Rd3GT1* and *Rd3GT6*), which may play a vital role in flower anthocyanin accumulation, were selected and further functionally characterized. Temporal and spatial expressions of *Rd3GT1* and *Rd3GT6* were detected and their potential roles *in planta* were examined *via* introducing into *Arabidopsis UGT78D2* mutant and *Nicotiana tabacum* plants. Furthermore, biochemical properties of *Rd3GT1* and *Rd3GT6* proteins were also confirmed. The results displayed that both flavonoid 3-*O*-glycosyltransferases performed the crucial roles on flower color formation as well as anthocyanin biosynthesis in *R. delavayi*. To our knowledge, this is the first report of the characterization of flavonoid 3-*O*-glycosyltransferase in *R. delavayi* and the results will not only provide new insights into the biosynthesis of anthocyanin in *R. delavayi* but also contribute to the further study of UTGs sugar donor preference and structure.

## Materials and Methods

### Plant Materials

*R. delavayi* plants were cultivated in the experimental field of National Forest Garden of GuiZhou Province, China. The roots, leaves, petals, pistils, stamens, toruses, scapes, and developing flowers (stages 1–5) were collected. Wild-type *Arabidopsis* (*Arabidopsis thaliana*) and T-DNA insertion mutant (*UGT78D2*) were purchased from the Nottingham Arabidopsis Stock Center (NASC) and maintained in a long day condition (16 h light/8 h dark photoperiod). For RT-PCR and anthocyanin analysis, *Arabidopsis* seedlings were harvested at 7 days after germination on 1/2 MS medium containing 3% sucrose (anthocyanin induction medium). *Nicotiana tabacum* plants used for transformation were grown in a glasshouse at 22°C with a 12 h light/12 h dark photoperiod, and full-blooming flowers of T1 transgenic *Nicotiana tabacum* were sampled for further analysis. All samples mentioned above were frozen immediately in liquid nitrogen, and kept at −80°C until further use.

### Chemicals

UDP-glucose, UDP-galactose, UDP-rhamnose, UDP-arabinose, cyanidin, delphinidin, and malvidin were purchased from Sigma-Aldrich (USA), and pelargonidin, petunidin, peonidin, cyanidin 3-*O*-glucoside, delphinidin 3-*O*-glucoside, pelargonidin 3-*O*-glucoside, malvidin 3-*O*-glucoside, petunidin 3-*O*-glucoside, and peonidin 3-*O*-glucoside were obtained from Phytolab (Germany).

### Detection of Anthocyanins

The extraction and detection of anthocyanins were conducted as previously described (Sun et al., 2016). Briefly, 0.3 g freeze-dried flowers of *R. delavayi* were pulverized in liquid nitrogen and extracted with H_2_O: MeOH: HCl (75/24/1v/v/v) overnight at 4°C in darkness. After centrifugation, the extracts were filtered (with 0.22 μm microporous membrane) and separated by an HPLC system (Shimadzu) equipped with an ACCHROM XUnion C18 column (250 mm × 4.6 mm, 5 μm). The absorbance of anthocyanin pigments was monitored at 520 nm, and the flow rate was 1 ml/min. Then, the mobile phase A (5% formic acid in H_2_O) and B (methanol) worked as follows: 0–10 min, 14–17% B; 10–35 min, 17–23% B; 35–60 min, 23–47% B; 60–67 min, 47–14% B; 67–70 min, 14% B. According to the procedures depicted by Sun et al. (2015) mass spectrometer was selected for qualitative analysis (Sun et al., 2015). The contents of anthocyanin in each sample were calculated *via* the external standard calibration of cyanidin 3-*O*-glucoside standards (Fanali et al., 2011). The calibration curves used were linear (*R*^2^ > 0.99) and the concentration ranges were 5–1,000 μg/ml. Mean values were obtained from three biological replicates per sample.

### Expression Profiles of *Rd3GTs* Using Real-Time PCR

Total RNA for real-time PCR was extracted from flowers and other vegetative tissues of *R. delavayi* using RNA pure Plant Kit (CWBIO, China). Synthesis of first strand cDNA from 1 μg total RNA was performed using oligo (dT) and M-MLV reverse transcriptase (Takara, Japan). Then, real-time detection was conducted by using BioRad CFX96 Real-Time PCR System (BIO-RAD, Hercules, CA, USA) and TransStart^®^ Green qPCR SuperMix (TRANSGEN, China) with primers designed based on sequence information from transcriptome analysis performed previously (Supplementary Table 1). Amplification of *RdActin* under the identical conditions was carried out to normalize the levels of *Rd3GTs* as follows: 60 s at 95°C, followed by 40 cycles of 5 s at 95°C and 60 s at 60°C. Each sample was carried out in three biological replicates, and the relative expression levels of target genes were calculated by formula 2^−Δ*ΔCt*^. Meanwhile, the specific amplification of each gene was confirmed by melting curve analysis and agarose gel electrophoresis.

### Cloning of *Rd3GT* Candidate Genes

According to the methods mentioned above, cDNAs synthesized from flower of *R. delavayi* were used as PCR templates. Specific primers obtained from the assembled transcriptomic information (Supplementary Table 1) were used to clone the full-length coding sequence of *Rd3GT1* and *Rd3GT6*. After PCR amplification, the products were sub-cloned into the T/A cloning vector pMD18-T (Takara, Japan), and transformed into *Escherichia coli* JM109 competent cells. After positive screening, the correct recombinant clone was verified by sequencing.

### Sequence Alignment and Phylogenetic Analysis

Alignment of amino acid sequences was carried out by using the DNAMAN 6.0 software. The multiple sequence alignment was performed through using Clustal Omega. Based on this alignment, a phylogenetic tree was drawn using MEGA5.1 with neighbor-joining method and 1,000 bootstrap replicates.

### Plant Transformation

For ectopic expression of *Rd3GTs* in *Arabidopsis* and *Nicotiana tabacum*, their full-length CDS were amplified from the pMD18-T vector and inserted into the binary vector pBI121 digested with *Xba* I and *BamH* I, generating the pBI121-*Rd3GTs* overexpression constructs. Then, the resulting constructs were confirmed by sequencing and introduced into *Agrobacterium tumefaciens* strain GV3101 for *Arabidopsis* and *Nicotiana tabacum* transformation. Subsequently, inflorescences of *Arabidopsis* mutant (*UGT78D2*) were transformed *via* floral dip method, as described by Clough (Clough and Bent, 1998). The harvested seeds were selected on 1/2 MS medium supplemented with 50 mg/l kanamycin to set T1 seeds. After growing the seeds on anthocyanin induction media (1/2 MS medium containing 3% sucrose) for 7 days, the T2 transgenic seedlings were collected and used for molecular and anthocyanin analysis. At the same time, *Nicotiana tabacum* transformation was also performed according to the method previously depicted by Sparkes (Sparkes et al., 2006). Through resistant selection, T1 transgenic *Nicotiana tabacum* seedlings were obtained and cultured in green house, and after flowering, their full-blooming flowers were used for sample collection and later on analysis. For confirming the expressions of *Rd3GTs*, RT-PCR analysis was conducted both in *Arabidopsis* and *Nicotiana tabacum*. Furthermore, total anthocyanin concentration in T2 transgenic *Arabidopsis* seedlings and T1 transgenic *Nicotiana tabacum* flowers was determined using the method described above with three biological replicates.

### Expression and Purification of *Rd3GTs* Proteins

The full-length CDS of *Rd3GT1* and *Rd3GT6* was introduced into the *EcoR* I/*EcoR* I and *BamH* I/*Hind* III site of pET32a (+) expression vector, respectively, and transformed into *E. coli* strain BL21 (DE3). On the second day, transformed *E. coli* expressing the recombinant *Rd3GTs* protein was cultured in liquid Luria-Bertani (LB) medium at 37°C till the value of OD_600_ of 0.6 was reached; after that, the cultures were induced several times for 36 and 48 h at 15°C through adding 0.2 mM of isopropyl-β-d-thiogalactopyranoside (IPTG). The cells were then collected by centrifugation (5,000 rpm, 10 min, 4°C) and suspended in phosphate-buffered saline without protein inhibitor (PBS, pH 7.4). After sonication in ice, the cell debris was removed and filtered with a 0.45 μm filter (Millipore). Next, the supernatant which contained soluble recombinant protein was purified *via* Ni-NTA pre-packed column (TransGen, China) and its purity was checked by sodium dodecyl sulfate polyacrylamide gel electrophoresis (SDS-PAGE). The concentration of recombinant *Rd3GTs* protein was measured by NanoDrop 1,000 Spectrophotometer (Thermo scientific, Waltham, MA, USA).

### Enzyme Assays

*In vitro* enzymatic activity of *Rd3GT1* and *Rd3GT6* was tested by using cyanidin and UDP-Glu as substrates. The assay reaction was carried out at 30°C for 5 min in a final volume of 200 μl containing 20–30 μl purified *Rd3GT1* and *Rd3GT6* protein, 100 mM potassium phosphate buffer (pH 8.0), 10 mM UDP-glucose, and 100 μM cyanidin. After quenching by adding 5% HCl (50 μl), the reaction mixture was centrifuged (12,000 rpm, 5 min, 4°C) and analyzed by HPLC using the method described above in “Detection of Anthocyanins”; then, the corresponding glucosylation product was confirmed by comparing it with the standard. Meanwhile, the protein extracted from BL21 (DE3) cells that express empty pET-32a (+) vectors was always used as a negative control. For determination, the substrate specificity of recombinant *Rd3GT1* and *Rd3GT6*, delphinidin, pelargonidin, petunidin, peonidin, and malvidin was selected as acceptors using UDP-glucose as a donor. In addition, 10 mM UDP-galactose, UDP-rhamnose, and UDP-arabinose were also used to examine the specificity of sugar donors.

### Statistical Analysis

Correlation analysis was carried out *via* calculating pairwise Pearson correlations between each *Rd3GT* gene and anthocyanin. *p* < 0.05 was taken as statistically significant by Student's *t*-test.

## Results

### Anthocyanin Profiling in Developing Flower

To uncover the detailed biochemical basis of the red color of *R. delavayi* flower, its anthocyanin profiles were identified and quantified during flower development by using HPLC (Figure 1A). The results showed that a total of eight peaks (A1–A8) were identified in flowers (Figure 1B). These eight peaks were then confirmed as delphinidin 3-*O*-galacoside, cyanidin 3-*O*-galacoside, delphinidin rhamnoside, cyanidin 3-*O*-glucoside, cyanidin 3-*O*-arabinoside, and cyanidin derivatives according to the MS analysis (Table 1). But pelargonidin glycosides, one kind of the basic anthocyanin, were not detected in *R. delavayi* flower. To correlate anthocyanin accumulation with gene expression, anthocyanin contents in five developmental stages of *R. delavayi* flower were determined. During flower development, anthocyanin accumulations declined gradually from stage 1 to stage 3 and raised at stage 4 with the minimum levels at stage 5 (Figure 1C). Interestingly, among different anthocyanins, anthocyanidin 3-*O*-glycoside was used most of the times, which accounted for 93.68–96.31% of the total anthocyanins (Figure 1D).

**Table 1:** The anthocyanin profiles in acidic MeOH-H_2_O extracts of the *R. delavayi*.

**Peak number**	**Identifacation/tentative identification**	**Retention time (min)**	**λmax (nm)**	**ESI-MS (m/z)**
A1	Delphinidin 3-galacoside	20.824	253	303[Dp+H]+
			511	465[M+H]+
A2	Cyanidin 3-galacoside	24.020	252	287[Cy+H]+
			521	449[M+H]+
A3	Delphinidin rhamnoside	25.310	255	303[Dp+H]+
			532	449[M+H]+
A4	Cyanidin 3-glucoside	26.283	255	287.1[Cy+H]+
			538	449[M+H]+
A5	Cyanidin 3-arabinoside	28.320	256	287.1[Cy+H]+
			531	419[M+H]+
A6	Other cyanidin derivatives	29.531	250	286.9[Cy+H]+
			523	419.1[M+H]+
A7	Other Cyanidin derivatives	34.221	249	287[Cy+H]+
			535	433.1[M+H]+
A8	Other Cyanidin derivatives	36.287	256	287.2[Cy+H]+
			516	419.1[M+H]+

### Selection of Candidate *Rd3GTs* From Flowers of *R. delavayi*

**Figure 2:**
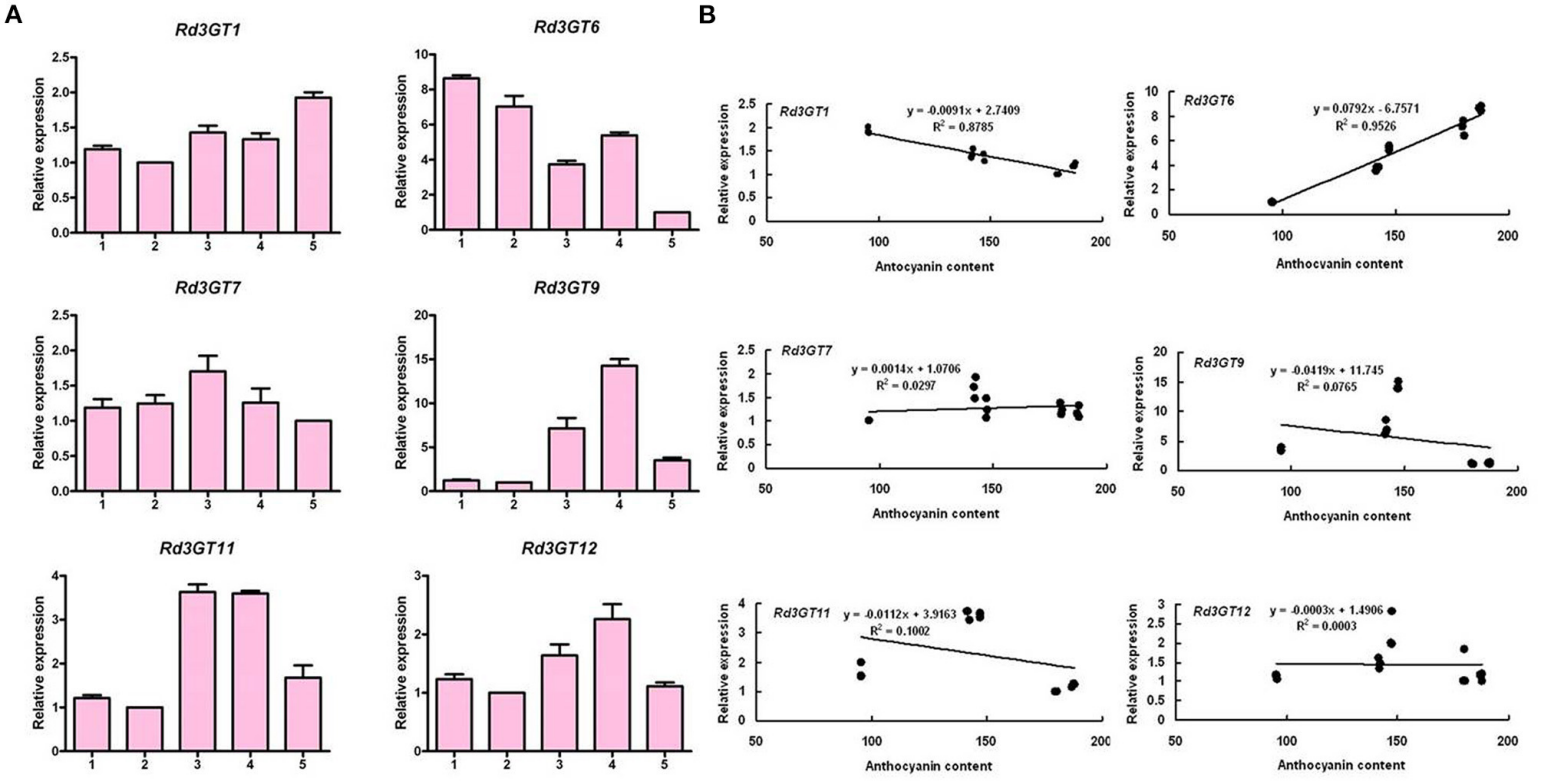
Expression profiles of *Rd3GTs* during flower development **(A)** and Pearson's correlation coefficient between *Rd3GTs* transcript levels and anthocyanin contents in *R. delavayi* flower **(B)**. 1–5 represent the flowers of different developmental stages.

On the basis of transcriptome data of different tissues of *R. delavayi*, a total of six potential *3GT* genes were identified through blastn alignment with reference genes from proximal species and *Arabidopsis*. To confirm the key *3GT* genes related to the biosynthesis of anthocyanins in *R. delavayi* flowers, we performed the expression analysis of these genes during flower development. As shown in Figure 2, transcript abundance of *Rd3GT1* (*R*^2^ = 0.8785) decreased from stage 1 to stage 2, followed by a raised transcript level to stage 5, and thus exhibited a negative correlation to the accumulation patterns of anthocyanin in *R. delavayi* flowers. For *Rd3GT6*, its transcripts (*R*^2^ = 0.9526) declined from stage 1 to stage 3, and significantly upregulated at stage 4 with minimum transcript levels noted at stages 5, which perfectly correlated with the anthocyanin accumulation profiles in *R. delavayi* flowers. But for *Rd3GT7* (*R*^2^ = 0.0297), *Rd3GT9* (*R*^2^ = 0.0765), *Rd3GT11* (*R*^2^ = 0.1002), and *Rd3GT12* (*R*^2^ = 0.0003), there was a weak or nearly no correlation between their expressions and anthocyanin accumulations. Therefore, *Rd3GT1* and *Rd3GT6* were preliminarily identified as the key *3GT* genes involved in the formation of *R. delavayi* flower color and selected for further functional characterization.

### Sequence and Phylogenetic Analyses

**Figure 3:**
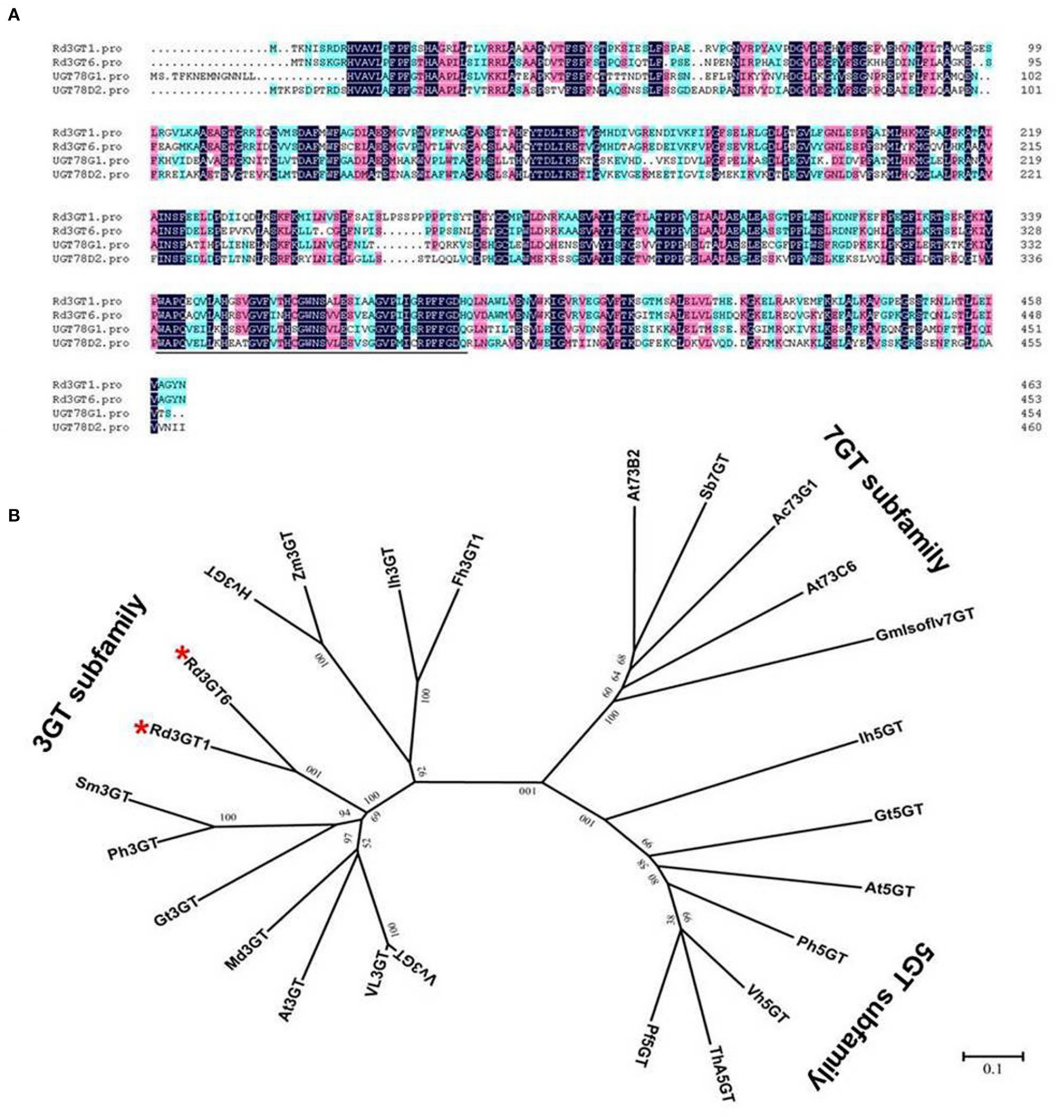
Sequence alignment and phylogenetic analyses of *Rd3GT1* and *Rd3GT6*. **(A)** Sequence alignment of *Rd3GT1* and *Rd3GT6* with *UGT78D2* (*Arabidopsis thaliana*, NM_121711.5) and *UGT78G1* (*Medicago sativa*, A6XNC6). The PSPG motif that interacts with the sugar donor is underlined. **(B)** Phylogenetic analyses of the deduced amino acids of *Rd3GT1* and *Rd3GT6* and UFGTs from different plant species. GenBank accession numbers are as follows: VvFd3GT (*Vitis vinifera* AAB81683), Vl3GT (*Vitis labrusca* ABR24135), Mt3GT (*Medicago truncatula* XP_003610163), At3GT (*Arabidopsis thaliana*, NM_121711.5), Gt3GT (*Gentiana triflora* Q96493.1), Ph3GT (*Petunia hybrida* AB027454), Sm3GT (*Solanum melongena* Q43641), Zm3GT (*Zea mays* CAA31856), Hv3GT (*Hordeum vulgare* CAA33729), Ih3GT (*Iris hollandica* BAD83701), Fh3GT1 (*Fressia hybrida* ADK75021.1), Ih5GT (*Iris hollandica* BAD06874), Gt5GT (*Gentiana triflora* BAG32255), At5GT (*Arabidopsis thaliana* NP_193146), Pf5GT (*Perilla frutescens* BAA36421), Th5GT (*Torenia hybrid* BAC54093), Ph5GT (*Petunia hybrida* AB027455), Vh5GT (*Verbena hybrida* AB076698), Sb7GT (*Scutellaria baicalensis* BAA83484), At73B2 (*Arabidopsis thaliana* NM_179161.2), At73C6 (*Arabidopsis thaliana* NM_129234.2), Ac73G1 (*Allium cepa* AAP88406), GmIsoflv7GT (Glycine max ABB85236.1). *Rd3GT1* and *Rd3GT6* are highlighted in red stars.

Based on sequence information of transcriptome data, the coding region sequences of *Rd3GT1* and *Rd3GT6* were successfully cloned from flower of *R. delavayi*. The ORFs of *Rd3GT1* and *Rd3GT6* were 1,395 and 1,365 bp long encoding 464 and 454 amino acids residues, respectively. Multiple sequence alignment of *Rd3GT1* and *Rd3GT6* with *UGT78G1* (flavonoid 3-*O*-glucosyltransferase from *Medicago truncatula*, A6XNC6) and *UGT78D2* (flavonoid 3-*O*-glucosyltransferase from *Arabidopsis thaliana*, NM_121711.5) revealed that both *Rd3GTs* carried the conserved 44-residue C terminal PSPG signature motif, and the last residue within this motif was histidine (Figure 3A). Next, a neighbor-joining phylogenetic tree of plant flavonoid UGTs was constructed, and three major clusters, which exhibited activities specific toward 3-OH, 5-OH, and 7-OH glycosylation, were recognized. *Rd3GT1* and *Rd3GT6* were grouped with 3-OH cluster implying that these two glucosyltransferases might belong to flavonoid 3-*O*-glycosyltransferase and glycosylate at the 3-OH of flavonoid substrates (Figure 3B).

### Expression Analysis of *Rd3GTs*

**Figure 4:**
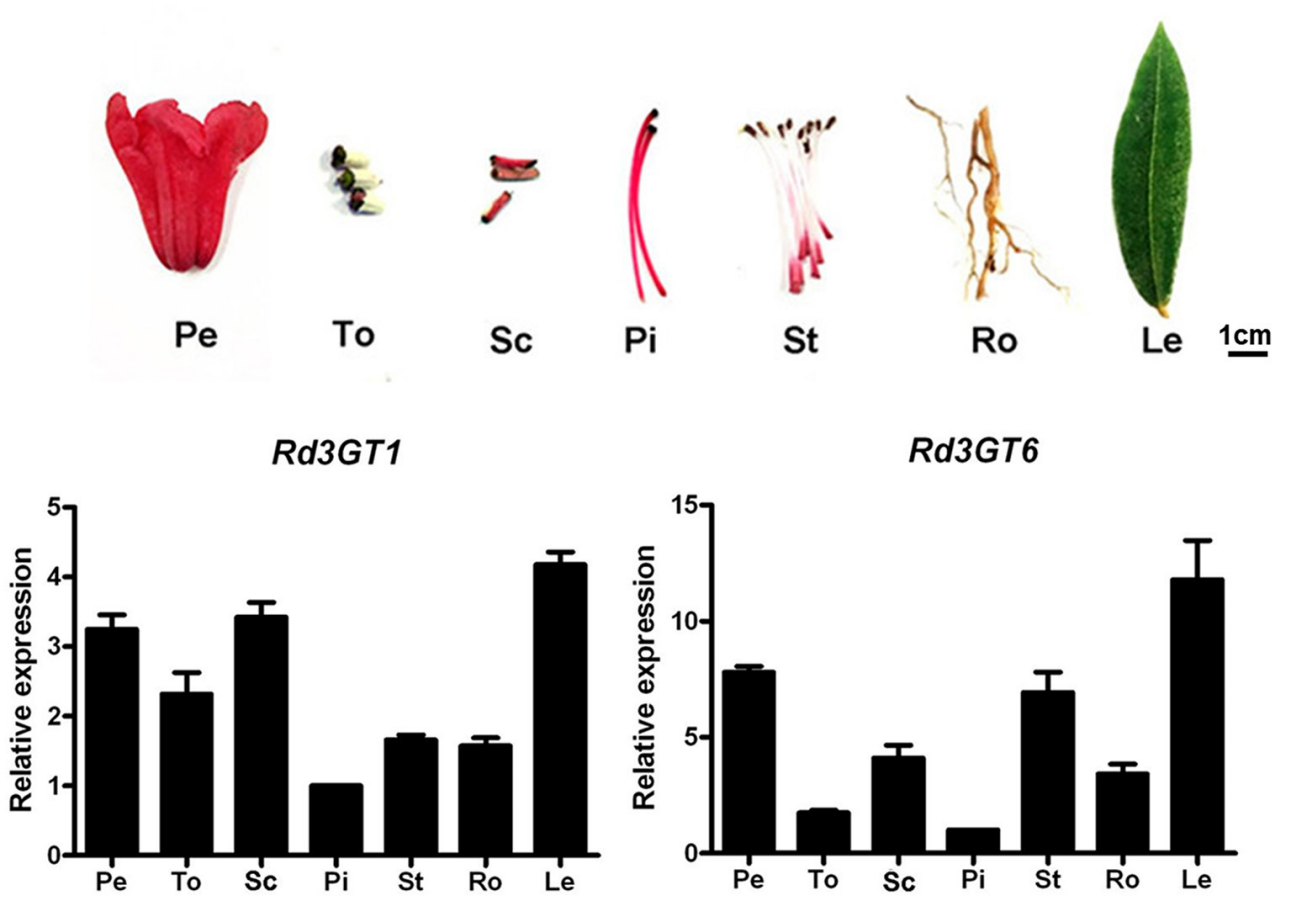
Expression profiles of *Rd3GT1* and *Rd3GT6* in different tissues of *R. delavayi*. Pe, petals; To, toruses; Sc, scapes; Pi, pistils; St, stamens; Ro, roots; Le, leaves.

Transcript levels of *Rd3GT1* and *Rd3GT6* were also determined in different *R. delavayi* organs by real-time qRT-PCR. As shown in Figure 4, *Rd3GT1* and *Rd3GT6* transcripts were detected in all organs tested, and their expressions were tissue specific. Accordingly, both *Rd3GT1* and *Rd3GT6* were most highly expressed in leaves where anthocyanins were barely detected (Supplementary Figure 4), and exhibited relatively lower expressions in pistil. Nevertheless, expression analysis during flower development displayed that the mRNA levels of *Rd3GT1* and *Rd3GT6* were dependent on flower development, and significantly correlated with the accumulation of anthocyanin. Taken together, this integrative expression analysis suggests that *Rd3GT1* and *Rd3GT6* may participate in not only the anthocyanin but also other flavonoid glycoside biosynthesis in *R. delavayi*.

### Functional Identification of *Rd3GTs* in Planta

**Figure 5:**
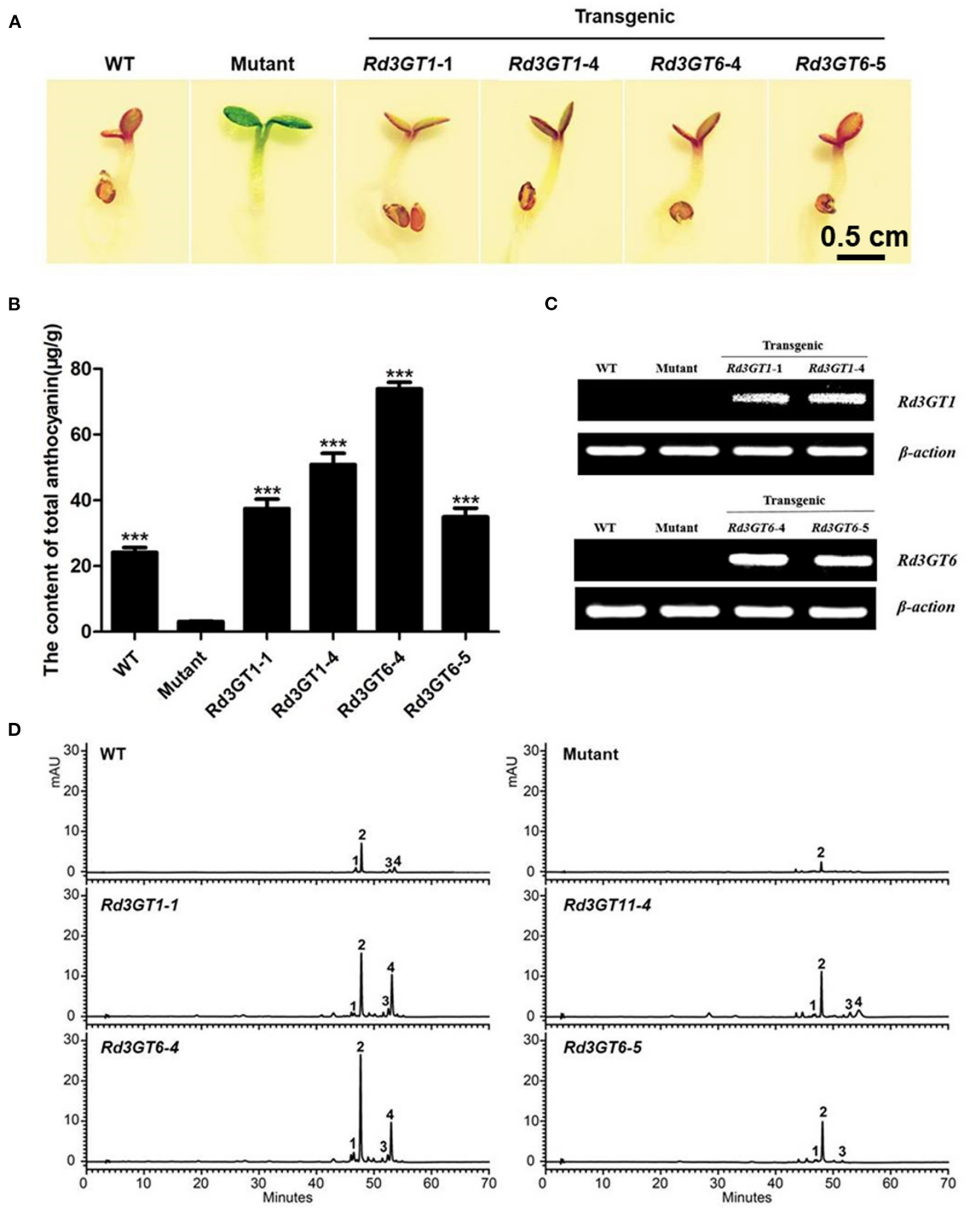
Complementation of the pigmentation of *UGT78D2* mutant seedlings with *Rd3GT1* and *Rd3GT6*. **(A)** Phenotypes of wild-type, mutant, and transgenic *Arabidopsis* seedlings. **(B)** Contents of anthocyanins in *Arabidopsis* seedlings. **(C)** Expressional analysis of *Rd3GT1* and *Rd3GT6* by reverse transcription polymerase chain reaction. **(D)** HPLC analyses of anthocyanins in *Arabidopsis* seedlings. Data correspond to means of three biological replicates. Asterisks indicate significant differences between means of mutant and wild-type as well as transgenic plants calculated by Student's *t*-test (^***^p < 0.001).

To investigate the function of *Rd3GT1* and *Rd3GT6 in vivo*, these two genes were overexpressed in the *Arabidopsis* mutant (*UGT78D2*), which failed to generate anthocyanin pigments in their cotyledon and hypocotyls. After resistance selection, seeds of the wild-type, *UGT78D2* mutant, and T2 transgenic plants were germinated and inductively cultured on 1/2 MS medium with 3% sucrose. As present in Figure 5A, the seedlings of *UGT78D2* mutant exhibited purple coloration while overexpressing *Rd3GT1* and *Rd3GT6*, although the mutant transformed with empty vector was still green (Supplementary Figure 5). Meanwhile, RT-PCR analysis was conducted for further confirming the expression of *Rd3GTs* in transgenic lines, and amplicons of expected size, which were absent in wild type and mutant were observed in transgenic plants (Figure 5C). In addition, extracted anthocyanin metabolites from the abovementioned seedlings were also analyzed by HPLC and HPLC-MS to determine the contents and kinds of individual anthocyanin (Supplementary Table 2). The results revealed that both *Rd3GT1* and *Rd3GT6* could restore the anthocyanin peaks (peak 1, 3, and 4) that lacked *UGT78D2* mutant (Figure 5D), and showed higher anthocyanin contents than that in wild-type as well as *UGT78D2* mutant (Figure 5B). Overall, these results confirmed that both *Rd3GT1* and *Rd3GT6* encode functional UF3GT protein that participated in the biosynthesis of anthocyanin in *Arabidopsis*.

**Figure 6:**
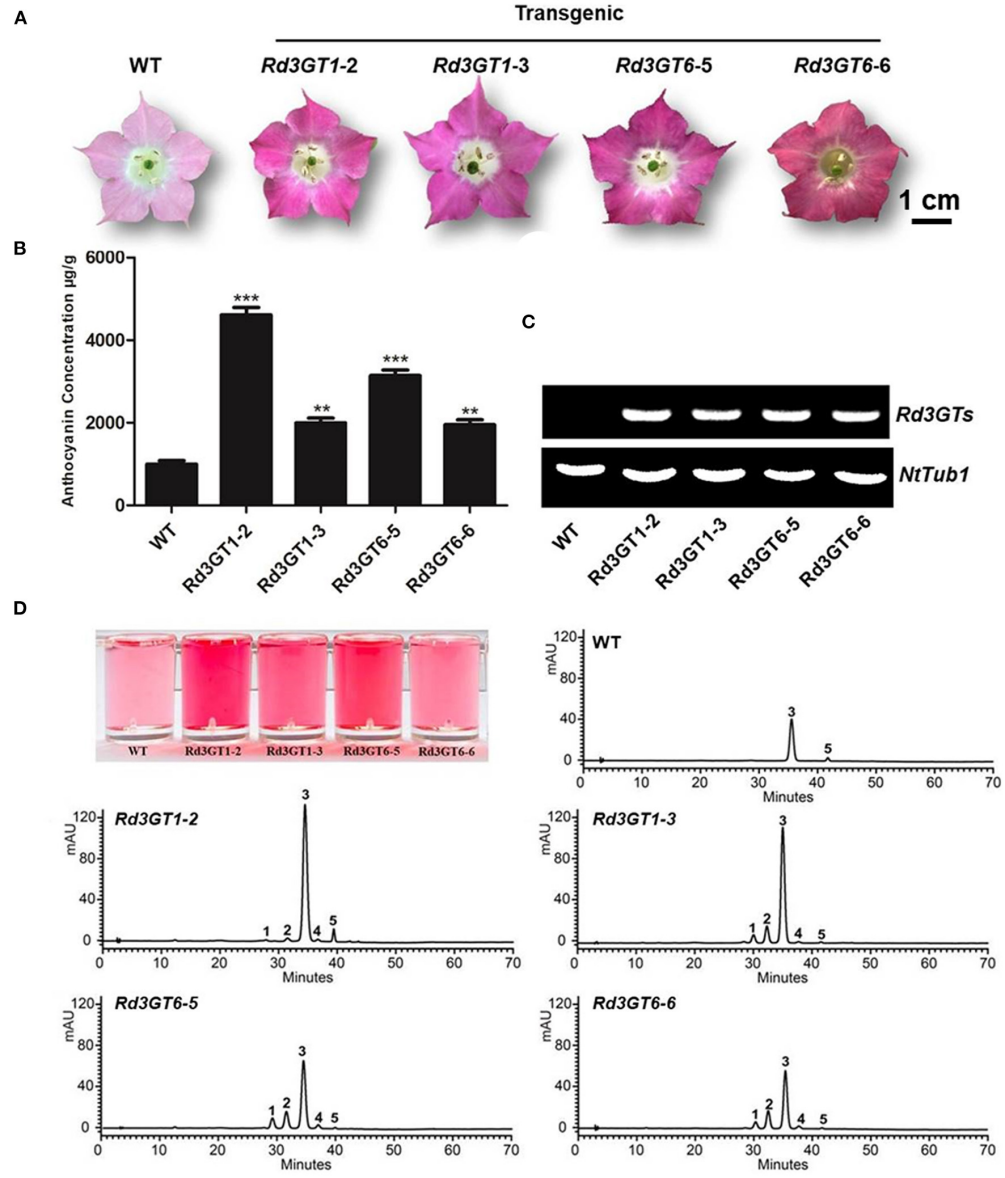
Effect of *Rd3GT1* and *Rd3GT6* on anthocyanin accumulation in transgenic tobacco flowers. **(A)** Tobacco flowers of wild-type and transgenic lines. **(B)** Contents of anthocyanin accumulation in transgenic tobacco flowers with HPLC. **(C)** Expression confirmation of *Rd3GT1* and *Rd3GT6* in flowers of transgenic tobacco. **(D)** HPLC analyses of anthocyanins in transgenic tobacco flowers. 1, Cyanidin 3-*O*-rutinoside 5-*O*-glucoside; 2, Cyanidin 3-*O*-arabinoside; 3, Cyanidin 3-*O*-rutinoside; 4, Cyanidin 3-*O*-xyloside; 5, Cyanidin 3-*O*-(6-*O*-malonyl-beta-D-glucoside). Results correspond to means from three biological replicates. Asterisks indicate significant differences between means of wild-type and transgenic plants calculated by Student's *t*-test (^**^p < 0.01); ^***^p < 0.001.

To further check the potential effect of *Rd3GT1*and *Rd3GT6* on plant flowers, a total of 24 transgenic tobacco plants overexpressing *Rd3GT1* and *Rd3GT6* were generated. Then, two independent transgenic lines were analyzed for each gene, and RT-PCR analysis was used to confirm the overexpression of *Rd3GT1* and *Rd3GT6* (Figure 6C). As shown in Figure 6A, flower color of transgenic plants was darker than the control. Next, for examining this color change in detail, anthocyanin was extracted from the corollas of transgenic lines and quantified using HPLC (Figure 6D). The results showed that anthocyanin levels in flowers of transgenic tobacco were all higher than those in wild type (Figure 6B). Simultaneously, the kinds of anthocyanin in transgenic flower were unexpectedly increased from 2 to 5 (Figure 6D), and these anthocyanins were subsequently identified as cyanidin 3-*O*-rutinoside 5-*O*-glucoside, cyanidin 3-*O*-arabinoside, cyanidin 3-*O*-rutinoside, cyanidin 3-*O*-xyloside, and cyanidin 3-*O*-(6-*O*-malonyl-beta-D-glucoside), respectively (Table 2). Thus, combining all these results, it becomes clear that the enzymes encoded by *Rd3GT1* and *Rd3GT6* can effectively increase the contents and kinds of anthocyanin in transgenic tobacco flower.

**Table 2:** HPLC-ESI-MS analysis of anthocyanin extracts of *Rd3GTs* over-expressing transgenic tobacco flowers.

**Peak number**	**Identifacation/tentative identification**	**Retention time (min)**	**ESI-MS (m/z)**
1	Cyanidin 3-*O*-rutinoside 5-*O*-glucoside	29.27	287.0
			657.2
2	Cyanidin 3-*O*-arabinoside	31.61	287.1
			419.1
3	Cyanidin 3-*O*-rutinoside	34.51	287.1
			595.2
4	Cyanidin 3-*O*-xyloside	36.75	287.1
			419.1
5	Cyanidin 3-*O*-(6-*O*-malonyl-beta-D-glucoside)	40.23	287.1
			535.1

### Biochemical Characterization of *Rd3GTs*

**Figure 7:**
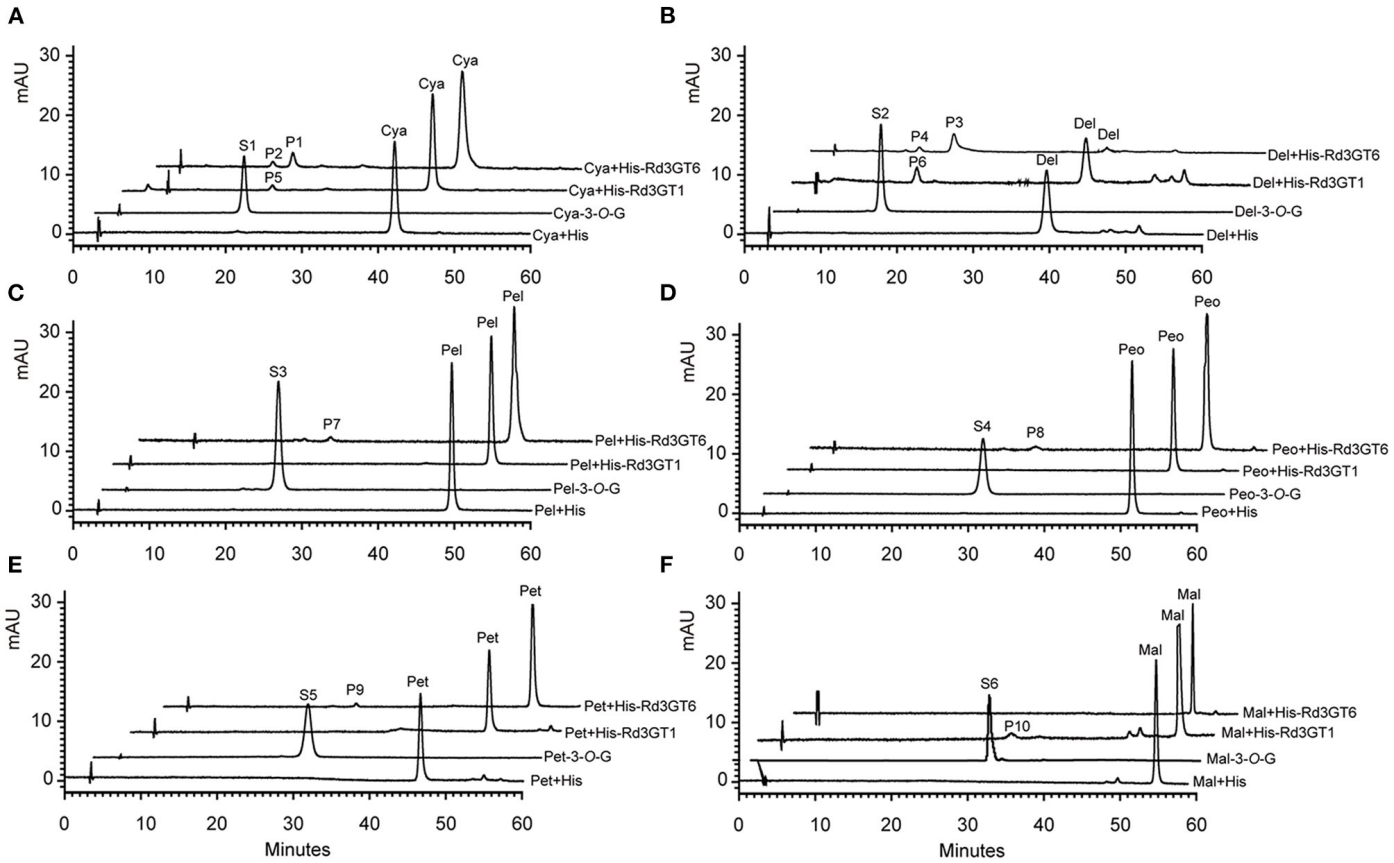
HPLC profiles of *Rd3GT1* and *Rd3GT6* reaction products with UDP-Glu and different anthocyanidin. **(A)** Cyanidin. P1/P5/S1, cyanidin 3-*O*-glucoside; P2, cyanidin 4'-*O*-glucoside, **(B)** Delphinidin. P3/P6/S2, delphinidin 3-*O*-glucoside; P4, delphinidin 4'-*O*-glucoside, **(C)** Pelargonidin. P7/S3, pelargonidin 3-*O*-glucoside, **(D)** Peonidin. P8/S4, peonidin 3-*O*-glucoside, **(E)** Petunidin. P9/S5, petunidin 3-*O*-glucoside, and **(F)** Malvinidin. P10/S6, malvinidin 3-*O*-glucoside. Cya-3-*O*-G, cyanidin 3-*O*-glucoside; Del-3-*O*-G, delphinidin 3-*O*-glucoside; Pel-3-*O*-G, pelargonidin 3-*O*-glucoside; Peo-3-*O*-G, peonidin 3-*O*-glucoside; Pet-3-*O*-G, petunidin 3-*O*-glucoside; Mal-3-*O*-G, malvinidin 3-*O*-glucoside.

The coding regions of *Rd3GT1*and *Rd3GT6* were cloned into pET-32a (+) vector, and introduced into an *E. coli* BL21 (DE3) strain. The recombinant soluble proteins of *Rd3GTs* were purified by Ni-NTA pre-packed column and analyzed by SDS-PAGE (Supplementary Figure 1). The function of *Rd3GT1* and *Rd3GT6* was characterized using cyanidin as the substrate and UDP-glucose as the sugar donor. HPLC analysis identified that both *Rd3GTs* could catalyze the transfer of glucose to the 3-OH of cyanidin *via* comparing it with the reference standard. The recombinant protein extracted from *E. coli* BL21 expressing the empty vector could not glucosylate cyanidin (Figure 7A). Then, the reaction conditions for *Rd3GTs* were optimized, and both *Rd3GTs* exhibited their maximum activity at pH 8.0 and 30°C, which has been applied in the analysis of most plant UFGTs (Masayuki et al., 2000; Kim et al., 2006).

To explore the substrate specificity of *Rd3GT*s, six anthocyanidin compounds were tested using UDP-glucose (UDP-Glu) as the sugar donor. *Rd3GT1* showed the highest conversion rates for delphinidin, whereas pelargonidin, peonidin, and petunidin could not be accepted by it. However, *Rd3GT6* was able to glucosylate five anthocyanidins except for malvinidin, and cyanidin exhibited the highest glucosylation rate, which was higher than that of *Rd3GT1* toward delphinidin (Supplementary Table 3). Furthermore, HPLC analysis of *Rd3GT6* reaction products showed that additional products (P2 and P4) were generated in the reaction of cyanidin and delphinidin (Figures 7A,B). The retention time of P1 and P3 was similar to the authentic cyanidin 3-*O*-glucoside and delphinidin 3-*O*-glucoside. Evidently, they were cyanidin 3-*O*-glucoside and delphinidin 3-*O*-glucoside, respectively. Then, HPLC-ESI-MS results demonstrated that the molecular weights of P1 and P2 were indistinguishable, indicating that only one UDP-glucose had been transferred to P2. It was reported that hypsochromic shift of UV spectra between *Rd3GT6* products and substrates could be used to confirm the regiospecificity of *Rd3GT6*. Glycosylation at both 3-OH and 4'-OH would produce a hypsochromic shift, while at 7-OH, it does not display any effect (Thomas et al., 1997; Kramer et al., 2003). Therefore, the results suggested that P2 was likely to be cyanidin 4'-*O*-glucoside, and P4 was likely to be delphinidin 4'-*O*-glucoside based on hypsochromic shift (Supplementary Table 4).

**Figure 8:**
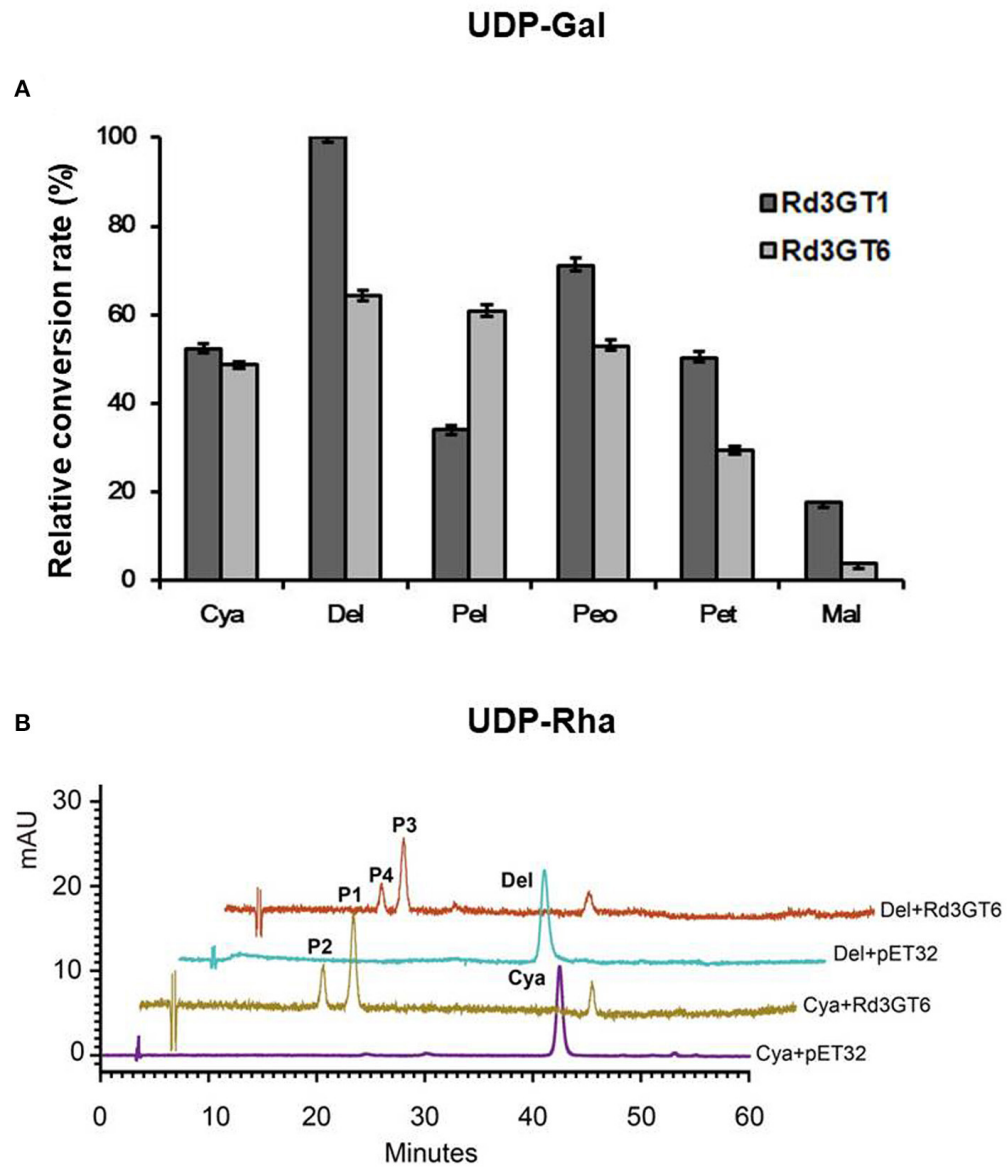
Relative activity of *Rd3GT1* and *Rd3GT6* toward several substrates and sugar donor. **(A)** Relative activity of *Rd3GT1* and *Rd3GT6* toward UDP- Gal and six anthocyanidins. The relative activity was calculated by *Rd3GT1* activity toward delphinidin as 100%. Cya, cyanidin; Del, delphinidin; Pel, pelargonidin; Peo, peonidin; Pet, petunidin; Mal, malvinidin. **(B)** HPLC profiles of *Rd3GT6* reaction products with UDP-Rha and cyanidin/delphinidin. P1, cyanidin 3-*O*-rhamnoside; P2, cyanidin 4'-*O*-rhamnoside; P3, delphinidin 3-*O*-rhamnoside; P4, delphinidin 4'-*O*-rhamnoside.

To further explore the sugar donor specificity of *Rd3GTs*, we tested three other donors, that is, UDP-galactose (UDP-Gal), UDP-rhamnose (UDP-Rha), and UDP-arabinose (UDP-Ara). Surprisingly, *Rd3GT6* exhibited high sugar donor promiscuity when catalyzing cyanidin, whereas *Rd3GT1* did not, because it could not transfer UDP-Rha and UDP-Ara (Supplementary Tables 5, 6). In the presence of UDP-Gal, both *Rd3GT1* and *Rd3GT6* were able to efficiently glycosylate all the anthocyanidin substrates (Figure 8A). And with regard to UDP-Rha, *Rd3GT6* could catalyze the glycosylation of cyanidin as well as delphinidin and showed relatively higher glycosylation rates toward delphinidin (Supplementary Table 6). For the catalytic reaction of UDP-Rha, *Rd3GT6* produced four products (P1–P4), and all these products were identified as mono-*O*-glycosides through HPLC-ESI-MS analysis; thus, P1, P3 and P2, P4 were tentatively characterized as 3-*O*-rhamnoside and 4'-*O*-rhamnoside by the hypsochromic shift (Figure 8B; Supplementary Table 7). As shown in Supplementary Figure 3, only cyanidin could accept UDP-Ara at conversion rates of 5.96%.

## Discussion

**Figure 9:**
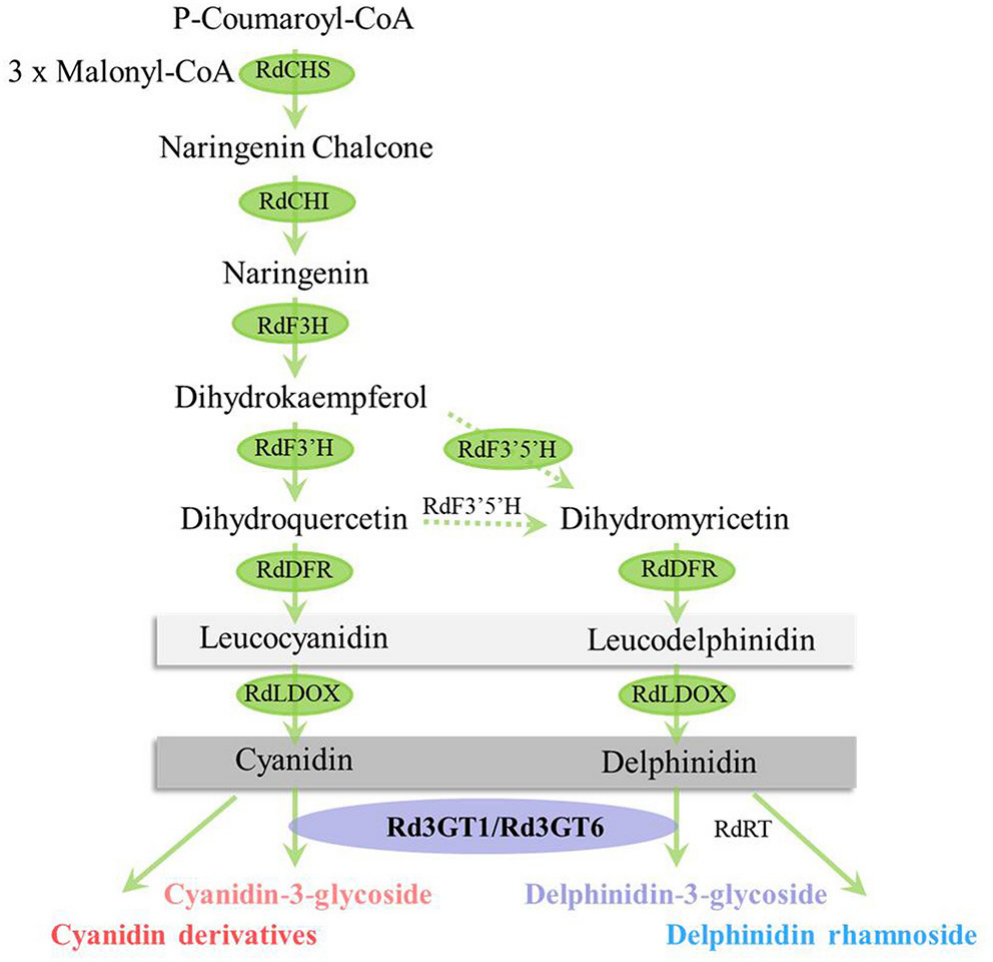
Proposed pathway leading to anthocyanin biosynthesis in the flowers of *R. delavayi*.

Combining tissue-specific expressions with an analysis of metabolites has been specifically proven as an efficient method to identify the function of gene. In this study, we first conducted the analysis of anthocyanin in developing flowers of *R. delavayi*, and according to the anthocyanins detected in flowers, biosynthetic pathway of anthocyanin in *R. delavayi* was proposed (Figure 9). As summarized in Table 1, only cyanidin and delphinidin aglycons accumulated in *R. delavayi*, while pelargonidin derivatives were undetectable. This result was in accordance with the study of grape hyacinth and *Cymbidium* (Johnson et al., 2010; Hongli et al., 2019), where absence of Pg-type anthocyanin was due to the DFR inability to reduce dihydrokaempferol (DHK). In support of this, we recently demonstrated that DFR1 of *R. delavayi* also could not catalyze DHK to form pelargonidin (Wei et al., 2021). On the contrary, quantitative analysis of anthocyanins showed that the level of anthocyanin gradually decreased during flower development, and anthocyanidin 3-*O*-glycoside always accounted for more than 93% at any developmental stage (Figure 1D). Thus, the occurrence of the large proportions of anthocyanidin 3-*O*-glycosides implies that flavonoid 3-*O*-glycosyltransferase must be crucial for *R. delavayi* flower color formation.

After correlation analysis between anthocyanin accumulations and *Rd3GTs* expressions, *Rd3GT1* and *Rd3GT6* were preliminarily defined as the pivotal *3GT* genes for anthocyanin formation in *R. delavayi* flowers (Figure 2B). Hence, their CDSs were successfully isolated from *R. delavayi* flowers, and phylogenetic analysis revealed that they were grouped into flavonoid 3-*O*-glycosyltransferase clade, which hinted the involvement of *Rd3GT1* and *Rd3GT6* in the 3-*O*-glycosylation of anthocyanidin in flowers of *R. delavayi* (Figure 3B). At the same time, multiple sequence alignment showed that these two *Rd3GTs* carried the typical PSPG sequence motif at the C terminal end, and their final amino acid residue within this motif was histidine (Figure 3A), which means that *Rd3GT1* and *Rd3GT6* are more likely to be galactosyltransferases (Akiko et al., 2004). Transcript analysis displayed that expressions of *Rd3GT1* and *Rd3GT6* were developmentally regulated. And among all *Rd3GTs, Rd3GT6* exhibited the strongest correlation with the accumulation of anthocyanins (Figure 2B), indicating that *Rd3GT6* might play a key role in anthocyanin biosynthesis during flower development. In addition, both *Rd3GT1* and *Rd3GT6* were also expressed in all the examined tissues, and not just the samples having anthocyanin (Figure 4). This expression profile is similar to *ANL1* of *Arabidopsis* (Kubo et al., 2004) and suggests that *Rd3GT1* and *Rd3GT6* perhaps glycosylate not only anthocyanidin but also other flavonoids.

Transfer of *Rd3GT1* and *Rd3GT6* into *Arabidopsis UGT78D2* mutant successfully restored the biosynthesis of anthocyanins in their cotyledons and hypocotyls (Figure 5A), which confirmed the functions of *Rd3GT1* and *Rd3GT6* as 3GT in planta. Similarly, *3GT* genes from black soybean and Freesia overexpressed in *UGT78D2* mutants also obtained the same results, hinting that 3GT proteins that take part in the biosynthesis of anthocynins are functionally exchangeable among diverse plants (Kovinich et al., 2010; Sun et al., 2016). Moreover, as shown in Figure 5D, pelargonidin derivatives (corresponding peak 3, Supplementary Table 2) which were absent in *R. delavayi* were detected in transgenic *Arabidopsis*; this observation suggests that both *Rd3GT1* and *Rd3GT6* could utilize pelargonidin as a substrate in *Arabidopsis*. For further investigating the potential role of *Rd3GT1* and *Rd3GT6* that participated in flower color development, they were introduced into tobacco plants. Comparing them to the wild type, transgenic tobacco plants expressing *Rd3GT1* and *Rd3GT6* generated deeper pink flowers (Figure 6A), and this alike phenomenon had also been observed in *Litchi chinensis* (Xiao et al., 2016). But surprisingly, introduction of *Rd3GT1* and *Rd3GT6* in tobacco synchronously led to the production of three novel anthocyanins in flowers, including cyanidin 3-*O*-rutinoside 5-*O*-glucoside, cyanidin 3-*O*-arabinoside, and cyanidin 3-*O*-xyloside (Table 2). Therefore, the abovementioned results apparently demonstrate that *Rd3GT1* and *Rd3GT6* are necessary for *R. delavayi* flower color development and can serve as useful molecular tools to improve the kinds of anthocyanin in plants.

Previously, it was reported that UGTs could recognize several different sugar donors, including UDP-Glu, UDP-Gal, UDP-Rha, UDP-Ara, UDP-Xyl, UDP-GlcUA, and so on. Of these donors, UDP-Glu is regarded as the most common sugar donor in plants (Sarah et al., 2008). Here, based on the kinds of anthocyanins detected in *R. delavayi* (Table 1), UDP-Glu, UDP-Gal, UDP-Rha, and UDP-Ara were selected to examine the UDP-sugar donor specificity of *Rd3GT1* and *Rd3GT6*. As shown in Supplementary Tables 5, 6, both *Rd3GT1* and *Rd3GT6* could catalyze the addition of UDP-Glu and UDP-Gal to anthocyanidin, and the glycosylation rates toward UDP-Gal are much higher than that toward UDP-Glu. These results demonstrate that *Rd3GT1* and *Rd3GT6* prefer UDP-Gal as their sugar donors, which are congruent with the discovery that the last amino acid of their PSPG box is histidine (Akiko et al., 2004). But at the same time, the use of UDP-Glu as a sugar donor implies that sugar donor recognition is very complex, and both multiple amino acids and other structural features are responsible for the determination of sugar specificity (Yonekura-Sakakibara et al., 2007). In addition, *Rd3GT6* also showed glycosylation activity to cyanidin and delphinidin when UDP-Rha and UDP-Ara act as sugar donors, but *Rd3GT1* could not. This suggests that *Rd3GT6* fulfills a much important role on determining the diversity of anthocyanins in *R. delavayi* flowers, which is consistent with its expression analysis. Meanwhile, it is interesting to note that when we use cyanidin and delphinidin as substrates, UDP-Glu and UDP-Rha act as sugar donors; apart from anthocyanidin 3-*O*-glycoside, anthocyanidin 4'-*O*-glycoside was also produced (Figures 7, 8). It has been reported that the presence of 3'-OH in acceptor molecules will influence the regiospecificity of glycosylation. If 3'-OH (e.g., cyanidin and delphinidin) is present, 4'-*O*-glycoside is preferentially generated. If it is missing (e.g., pelargonidin), then 7-*O*-glycoside is produced (Isayenkova et al., 2006). Perhaps, the production of 4'-*O*-glycoside for *Rd3GT6* toward cyanidin and delphinidin is due to this fact.

To further biochemically characterize *Rd3GT1* and *Rd3GT6*, different anthocyanidins were used as substrates to test their activity. As presented in Table 1, cyanidin glycoside and cyanidin derivatives were the predominant anthocyanins detected in *R. delavayi* flowers, suggesting that cyanidin might be the most efficient substrate for *Rd3GT1* or *Rd3GT6*. Indeed, on the one hand, *Rd3GT6* had a clear preference for cyanidin and could catalyze the transfer of all selected UDP-sugars to cyanidin (Supplementary Table 6). But, on the other hand, when UDP-Gal act as a sugar donor, the glycosylation efficiency of delphinidin was higher than that of cyanidin, which was conflicting to the relatively low levels of delphinidin glycoside in *R. delavayi* flowers (Supplementary Table 6). This similar phenomenon has also been observed from 3GT of *Medicago truncatula*, and revealed that UFGTs that take part in the biosynthesis of natural product are promiscuous, and also, the relative concentrations of substrates might be an important factor to determine their activity in planta (Modolo et al., 2007; Peel et al., 2009). Moreover, the substrate specificity analysis indicated that *Rd3GT6* could catalyze the addition of UDP-Glu and UDP-Gal to the 3-OH of pelargonidin *in vitro*, while pelargonidin 3-*O*-glycoside was undetected in *R. delavayi* flowers. This implies that *Rd3GT6* shows narrower substrate recognition *in vivo*, and biosynthesis of pelargonidin is stopped before *Rd3GT6*. Additionally, further research on UGTs should be conducted to explore how narrow vs. broad substrate recognition is regulated.

In summary, we first investigated the composition and contents of anthocyanins in *R. delavayi* flowers and proposed a pathway for its biosynthesis. Meanwhile, quantitative analysis of anthocyanins indicated that anthocyanidin 3-*O*-glycosides were the most prevalent at any flower development stage, suggesting that flavonoid 3-*O*-glycosyltransferase must play a vital role in flower color formation. Then, using a combined anthocyanin analysis and tissue-specific expressions approach, *Rd3GT1* and *Rd3GT6* were preliminarily confirmed as the key *3GT* genes for *R. delavayi* anthocyanin formation. Indeed, subsequent biochemical characterizations together with *in vivo* data demonstrated their significance on the biosynthesis of anthocyanins in *R. delavayi* flower. Moreover, *Rd3GT6* displayed great potential in sugar donor promiscuity and low regiospecificity, implying that it would be an attractive enzyme to engineer the diversity of anthocyanins for altering the color of plants and producing desired compounds. In conclusion, our findings make an important step forward in understanding the biosynthesis of anthocyanin in *R. delavayi* and will help to develop a useful method toward diversifying certain flavonoids in plants.

## Data Availability Statement

The original contributions presented in the study are included in the article/Supplementary Materials, further inquiries can be directed to the corresponding author/s.

## Author Contributions

ZJ and YY conceived and designed the study. WS, SS, HX, YC, and YW performed experiments, analyzed and interpreted the data, and wrote the manuscript. WS, XX, ZJ, and YY revised the manuscript critically. All authors read and approved the final manuscript.

## Funding

This work was supported by the National Natural Science Foundation of China (Grant No. 31760076), the Joint Fund of the National Natural Science Foundation of China and the Karst Science Research Center of Guizhou province (U1812401), grant from department of education of Guizhou Province (KY [2021]059), and grants from Guizhou Science and Technology project ([2017]5726 and [2019]1019).

## Conflict of Interest

The authors declare that the research was conducted in the absence of any commercial or financial relationships that could be construed as a potential conflict of interest.

## Publisher's Note

All claims expressed in this article are solely those of the authors and do not necessarily represent those of their affiliated organizations, or those of the publisher, the editors and the reviewers. Any product that may be evaluated in this article, or claim that may be made by its manufacturer, is not guaranteed or endorsed by the publisher.

## Abbreviations

HPLC: High-performance liquid chromatography
HPLC-MS: High-performance liquid chromatography-tandem mass spectrometry
IPTG: isopropyl-β-d-thiogalactopyranoside
SDS-PAGE: sodium dodecyl sulfate polyacrylamide gel electrophoresis
UDP-glucose: UDP-Glu
UDP-galactose: UDP-Gal
UDP-rhamnose: UDP-Rha
UDP-arabinose: UDP-Ara.
