# Supplementary material for: Characterization of Two Key Flavonoid 3-O-Glycosyltransferases Involved in the Formation of Flower Color in Rhododendron Delavayi
Source: Front Plant Sci. 2022 May 16;13:863482. doi: 10.3389/fpls.2022.863482 (PMC9149423; doi:10.3389/fpls.2022.863482)

Supplementary Material

SUPPLEMENTARY TABLES AND FIGURES

Supplementary Table S1 List of primers used in this study

Primer name	Primer sequence (5'→3')
Rd3GT1F-realtime	TCGGCGATCATCAGCTAAAC
Rd3GT1R-realtime	GACATTGTGCCACTCTTTGTG
Rd3GT6F-realtime	CACTTGCGGCTCATTGTTATAC
Rd3GT6R-realtime	CGGGACGAATTTCACGATTTC
Rd3GT7F-realtime	AGGGCGGAAACTTCACTAAAT
Rd3GT7R-realtime	CCATCACTTCCCACAGCTATC
Rd3GT9F-realtime	CTGGCTGAGAGGAAGAGTTATG
Rd3GT9R-realtime	GACCTATGCTCCAAGCCTTTA
Rd3GT11F-realtime	CCTGTTTGGAAACCTGGAATCT
Rd3GT11R-realtime	AGATCTTCGAAGGAGAGGATGG
Rd3GT12F-realtime	GGAACAGCGGAGAGTTGATAA
Rd3GT12R-realtime	TCCCATGACGTTCTTGTAGTG
Rd3GT1F-gene clone	TGACACCCACTTGGATAGTTTAG
Rd3GT1R- gene clone	CGACCTCTGGAATTGGATCAG
Rd3GT6F- gene clone	CTTTTCTCGCCGCTAAATG
Rd3GT6R- gene clone	TTTCTGCCACCTTCTCTG
Rd3GT1F-121	GCTCTAGAATGACCAAAAATATCTCA
Rd3GT1R-121	CGGGATCCCTAAAGATTGTACCCTGC
Rd3GT6F-121	GCTCTAGAATGACCAATTCCTCAAAA
Rd3GT6R-121	CGGGATCCCTAAAGGTTGTACCCTGC
Rd3GT1F-32	CGGAATTCATGACCAAAAATATCTCA
Rd3GT1R-32	CGGAATTCATGACCAAAAATATCTCA
Rd3GT6F-32	CGGGATCCATGACCAATTCCTCAAAA
Rd3GT6R-32	CCCAAGCTTCTAAAGGTTGTACCCTGC
NtTubA1-qRT-F	CTCCTATGCTCCTGTCATTTC
NtTubA1-qRT-R	GGCGAGGATCACACTTAAC
Atactin1-qRT-F	GGCCGTGGTAGTGAACATATAA
Atactin1-qRT-R	GTATGCCCTTCCTCATGCTATC
Rdactin1-qRT-F	GTATGCCCTTCCTCATGCTATC
Rdactin1-qRT-R	GGCCGTGGTAGTGAACATATAA

* Restriction enzyme site are underlined

Supplementary Table S2 HPLC-DAD and HPLC-ESI-MS analysis of anthocyanin extracts of the wild-type Arabidopsis and Rd3GTs over-expressing lines

Peak number	Identifacation/tentative identification	Retention time (min)	λmax (nm)	ESI-MS (m/z)	References
1	Cyanidin 3-*O*-[2″-*O*-(xylosyl) 6″-*O*-(*p*-*O*-(glucosyl) *p*-coumaroyl) glucoside] 5-*O*-[6′′′′-*O*-(malonyl) glucoside]	47.276	265<br>530	287.0<br>1137.2	Takayuki Tohge, Yasutaka Nishiyama et al., 2005 [1]
2	Cyanidin 3-*O*-[2″-*O*-(6‴-*O*-(sinapoyl) xylosyl) 6″-*O*-(p-*O*-(glucosyl)-*p*-coumaroyl) glucoside] 5-*O*-(6′′′′-*O*-malonyl) glucoside	48.573	257<br>532	287.1<br>1343.1	Stephen J. Bloora, Sharon Abrahamsb., 2002 [2]
3	Pelargonidin derivatives	53.851	277<br>525	271.2	
4	Cyanidin derivatives	54.998	275<br>532	287.1	

Reference list for Table S2

1. Tohge T, Nishiyama Y, Hirai MY, Yano M, Nakajima J, Awazuhara M, Inoue E, Takahashi H, Goodenowe DB, Kitayama M, Noji M, Yamazaki M, Saito K (2005) Functional genomics by integrated analysis of metabolome and transcriptome of *Arabidopsis* plants over-expressing an MYB transcription factor. Plant J 42: 218-235.
2. Stephen J. Bloor[a,] Sharon Abrahams[b] (2002) The structure of the major anthocyanin in Arabidopsis thaliana. Phytochemistry 59: 343-346.

Supplementary Table S3 Substrate specificity of recombinant Rd3GT1 and Rd3GT6

Substrates	Relative activity (%)	
	Rd3GT1	Rd3GT6
cyanidin	36.01±1.26	100[a]
delphinidin	53.01±3.39	50.68±3.19
pelargonidin	ND	40.78±1.77
peonidin	ND	90.98±0.89
petunidin	ND	37.57±2.47
malvinidin	40.30±3.67	ND

ND not detected due to the low activity

[a]The relative activity was calculated by Rd3GT6 activity toward cyanidin as 100%.

[b]The reactions were performed with UDP-glucose as the sugar donor.

Activity values are the mean of three independent determinations.

Supplementary Table S4 Structure assignment of Rd3GT6 reaction products towards UDP-glucose

Compounds	Retention time (min)	UV (nm)	Structure assignment
Cyanidin	42.11	254,525	Cyanidin aglycon
S1	19.61	254,514	Cyanidin 3-*O*- glucoside
P1[a]	19.47	254,515	Cyanidin 3-*O*- glucoside
P2[b]	16.44	255,516	Cyanidin 4'-*O*- glucoside
Delphinidin	39.54	253,530	Delphinidin aglycon
S2	14.04	252,521	Delphinidin 3-*O*-glucoside
P3[a]	14.15	251,520	Delphinidin 3-*O*- glucoside
P4[b]	12.33	251,521	Delphinidin 4'-*O*- glucoside

[a]The reaction products were assigned according to comparison of HPLC retention time and UV spectra with authentic compounds

[b]The reaction products were assigned according to the hypsochromic shift

Supplementary Table S5 Sugar donor specificity of recombinant Rd3GT1

Substrates	Relative activity (%)					
	cyanidin	delphinidin	pelargonidin	peonidin	petunidin	malvinidin
UDP-glucose	4.24±0.13	6.25±0.38	ND	ND	ND	4.75±0.45
UDP-galactose	52.37±1.08	100[a]	33.95±0.89	71.00±1.79	50.38±1.37	17.58±0.15
UDP-rhamnose	ND	ND	ND	ND	ND	ND
UDP-arabinose	ND	ND	ND	ND	ND	ND

Supplementary Table S6 Sugar donor specificity of recombinant Rd3GT6

Substrates	Relative activity (%)					
	cyanidin	delphinidin	pelargonidin	peonidin	petunidin	malvinidin
UDP-glucose	11.79±0.51	5.97±0.39	4.80±0.19	10.72±0.07	4.43±0.29	ND
UDP-galactose	48.90±0.38	64.21±1.30	60.73±1.50	53.01±1.37	29.40±0.83	3.72±0.14
UDP-rhamnose	5.19±0.31	8.49±0.42	ND	ND	ND	ND
UDP-arabinose	5.96±0.35	ND	ND	ND	ND	ND

ND not detected due to the low activity

[a]The relative activity was calculated by Rd3GT1 activity toward delphinidin as 100%.

Activity values are the mean of three independent determinations.

Supplementary Table S7 Structure assignment of Rd3GT6 reaction products towards UDP-rhamnose

Compounds	Retention time (min)	UV (nm)	Structure assignment
Cyanidin	42.37	254,524	Cyanidin aglycon
P1	19.80	253,514	Cyanidin 3-*O*- rhamnoside
P2	17.09	254,513	Cyanidin 4'-*O*- rhamnoside
Delphinidin	34.13	255,531	Delphinidin aglycon
P3	18.24	253,521	Delphinidin 3-*O*- rhamnoside
P4	16.02	252,520	Delphinidin 4'-*O*- rhamnoside

The reaction products were assigned according to the hypsochromic shift

Supplementary Figure S1. Expression of Rd3GTs in *E. coli.* (1) Maker (2) Total soluble protein from E. coli expressing pET-32a (+) vector (3) A total of 36 or 48 h after induction (4) Purified Rd3GTs.

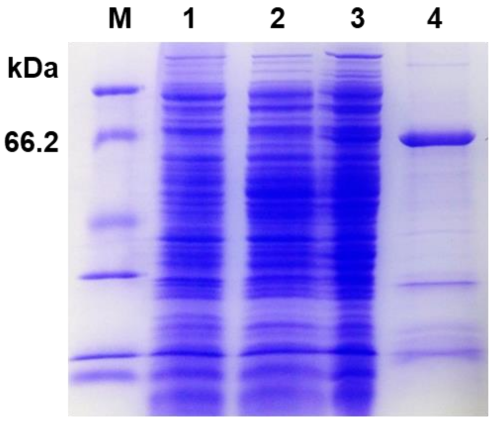

Rd3GT1

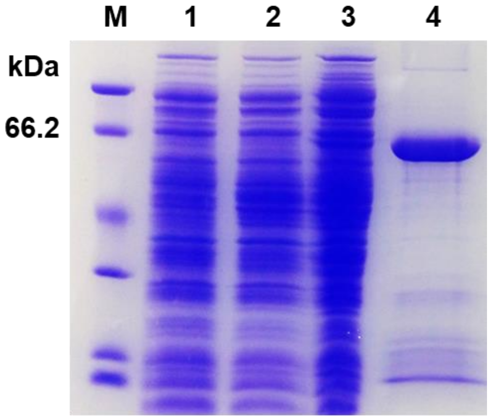

Rd3GT6

Supplementary Figure S2. HPLC profiles of Rd3GT1 and Rd3GT6 reaction products with UDP-Gla and different anthocyanidin.

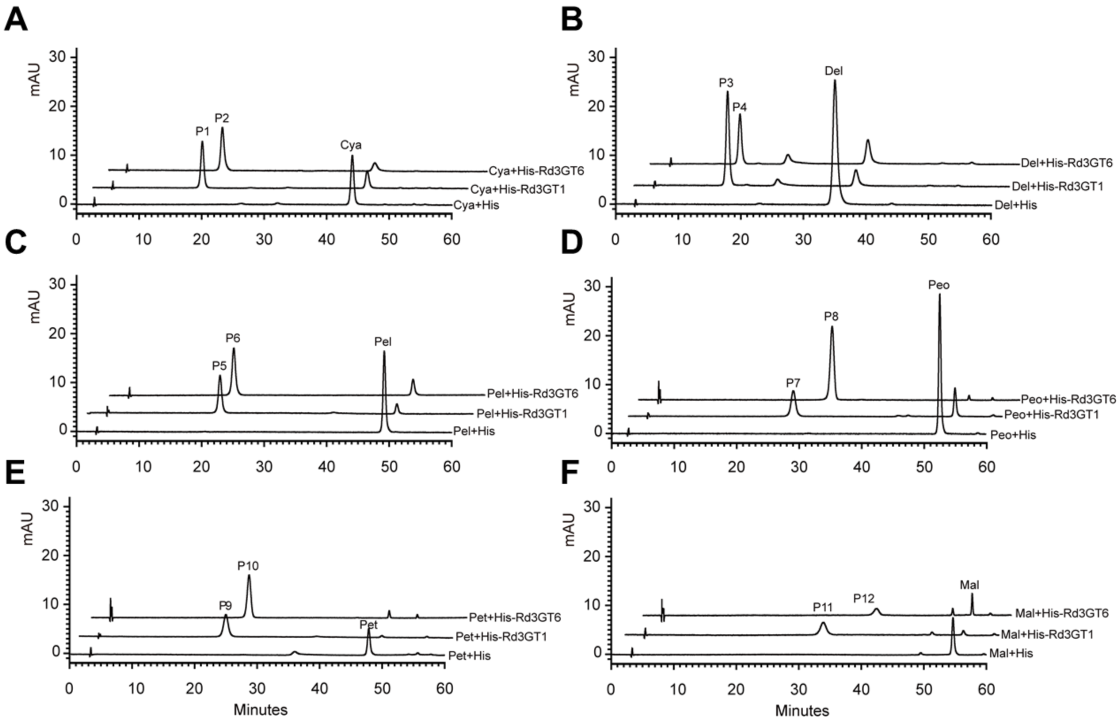

Supplementary Figure S3. HPLC profiles of Rd3GT6 reaction products with UDP-Ara and cyanidin.

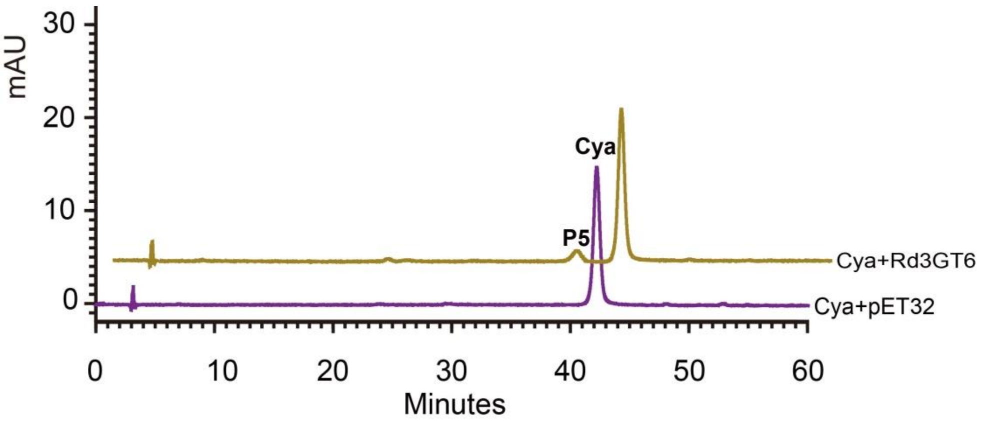

P5 indicated cyanidin 3-*O*-arabinoside

Supplementary Figure S4. Quantitative analyses of total anthocyanins in different tissues.

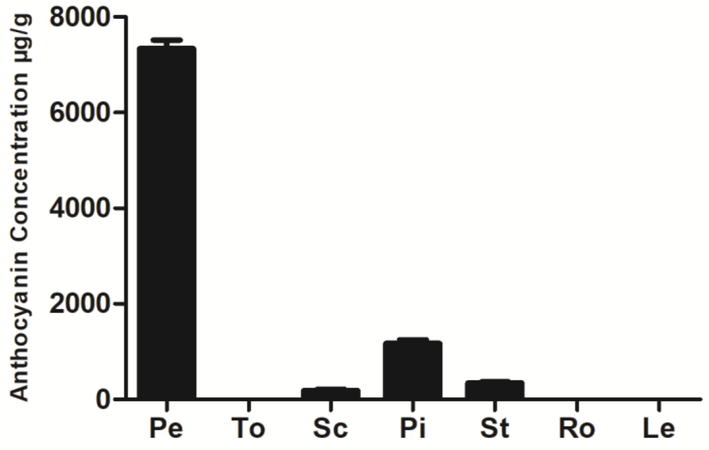

Supplementary Figure S5. Phenotypes of transgenic *Arabidopsis* seedlings transformed with empty vector.

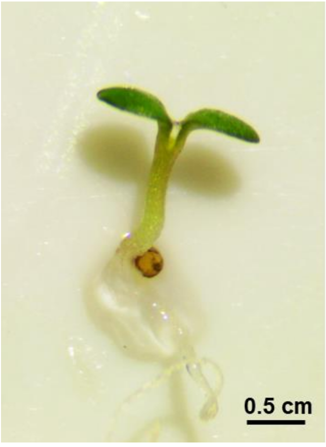

Supplementary Figure S6. Expression profiles of *Rd3GT1* and *Rd3GT6* in transgenic *Arabidopsis* seedlings.

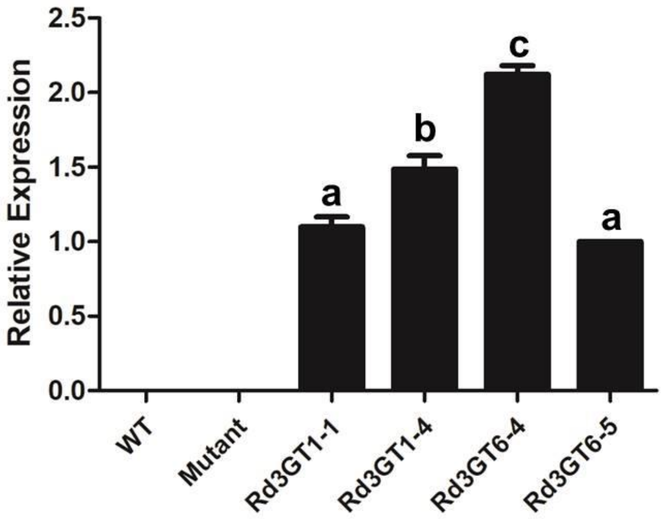

Supplement: Supplementary file 1 [file Data_Sheet_1.PDF]
